# Experimental assessment of pro-lymphangiogenic growth factors in the treatment of post-surgical lymphedema following lymphadenectomy

**DOI:** 10.1186/bcr2638

**Published:** 2010-09-08

**Authors:** Amy Baker, Harold Kim, John L Semple, Dan Dumont, Molly Shoichet, Dalia Tobbia, Miles Johnston

**Affiliations:** 1 Department of Laboratory Medicine and Pathobiology, University of Toronto, 27 King's College Circle, Toronto, Ontario M5S 1A1, Canada; 2 Sunnybrook Health Sciences Center, 2075 Bayview Avenue, Toronto, Ontario M4N 3M5, Canada; 3 Department of Surgery, Women's College Hospital, Grenville Street, Toronto, Ontario M5S 1B2, Canada; 4 Molecular and Cell Biology, Department of Medical Biophysics, University of Toronto, 27 King's College Circle, Toronto, Ontario M5S 1A1, Canada; 5 Department of Chemical Engineering & Applied Chemistry, Department of Chemistry, Institute of Biomaterials & Biomedical Engineering, University of Toronto, 27 King's College Circle, Toronto, Ontario M5S 1A1, Canada; 6 Department of Trauma, Plastic and Reconstructive Surgery, University Medicine, Robert-Koch-Strasse 40, D-37075 Göttingen, Germany

## Abstract

**Introduction:**

Lymphedema is a frequent consequence of lymph node excision during breast cancer surgery. Current treatment options are limited mainly to external compression therapies to limit edema development. We investigated previously, postsurgical lymphedema in a sheep model following the removal of a single lymph node and determined that autologous lymph node transplantation has the potential to reduce or prevent edema development. In this report, we examine the potential of lymphangiogenic therapy to restore lymphatic function and reduce postsurgical lymphedema.

**Methods:**

Lymphangiogenic growth factors (vascular endothelial growth factor C (VEGF-C)) and angiopoietin-2 (ANG-2) were loaded into a gel-based drug delivery system (HAMC; a blend of hyaluronan and methylcellulose). Drug release rates and lymphangiogenic signaling in target endothelial cells were assessed *in vitro *and vascular permeability biocompatibility tests were examined *in vivo*. Following, the removal of a single popliteal lymph node, HAMC with the growth factors was injected into the excision site. Six weeks later, lymphatic functionality was assessed by injecting ^125^Iodine radiolabeled bovine serum albumin (^125^I-BSA) into prenodal vessels and measuring its recovery in plasma. Circumferential leg measurements were plotted over time and areas under the curves used to quantify edema formation.

**Results:**

The growth factors were released over a two-week period *in vitro *by diffusion from HAMC, with 50% being released in the first 24 hr. The system induced lymphangiogenic signaling in target endothelial cells, while inducing only a minimal inflammatory response in sheep. Removal of the node significantly reduced lymphatic functionality (nodectomy 1.9 ± 0.9, HAMC alone 1.7 ± 0.8) compared with intact groups (3.2 ± 0.7). In contrast, there was no significant difference between the growth factor treatment group (2.3 ± 0.73) and the intact group indicating improved function with the molecular factors. An increase in the number of regenerated lymphatic vessels at treatment sites was observed with fluoroscopy. Groups receiving HAMC plus growth factors displayed significantly reduced edema (107.4 ± 51.3) compared with nontreated groups (nodectomy 219.8 ± 118.7 and HAMC alone 162.6 ± 141).

**Conclusions:**

Growth factor therapy has the potential to increase lymphatic function and reduce edema magnitude in an animal model of lymphedema. The application of this concept to lymphedema patients warrants further examination.

## Introduction

Lymphedema is a common complication of surgical breast cancer treatment, developing in 20% to more than 90% of patients, depending on the assessment criteria employed [1]. If left untreated, the edema can lead to recurring infections, impaired limb function, psychosocial problems and, in extreme cases, malignant complications and life-threatening infections. Most affected individuals are offered some form of nonsurgical external compression therapies to limit edema development, but there is great interest in developing more effective treatment options. One of the main problems, however, is that we do not understand clearly why lymphedema develops.

The pathogenesis of lymphedema is undoubtedly very complex, with multiple factors likely contributing to the development of chronic edema. In a previous report, we argued that the lymph node itself may have an important role in tissue fluid balance and observed that vascularized autologous lymph node transplantation could enhance lymphatic function and reduce edema significantly [2]. In addition, however, one must consider the damage to the lymphatic vessels that ensues when lymph nodes are removed. While lymphatics normally have an impressive capacity to regenerate following injury, it is possible that this process fails to compensate fully and that nonoptimal lymph transport conditions predispose the patient to edema formation.

With this in mind, various groups have investigated whether induction of lymphangiogenesis in animal models can impact lymphedema [3-6]. While the results are generally positive, the use of relatively small animals for these studies limits the amount of physiological information that can be acquired. One advantage of using sheep is that lymphatic function can be quantified relatively easily, and any potential interventions are more human-sized in their perspective [2,7].

In the experiments under consideration here, our objective was to test the principle that the delivery of lymphangiogenic growth factors into the nodal excision site would enhance vessel regeneration, reestablish lymph transport capabilities and reduce edema formation. In terms of identifying which factors to inject, vascular endothelial growth factor C (VEGF-C) seemed like an obvious choice, given its role in regulating new lymphatic vessel growth [8,9]. Another factor that we decided to introduce into the lesion site was angiopoietin-2 (ANG-2), which appears to play a role in the maturation of newly formed lymphatic networks [10].

Having determined the animal model and the therapeutic molecules to deliver, we then focused on the optimal delivery strategy. Osmotic minipumps or adenoviral vectors are commonly employed to introduce molecules into the tissues. However, an evolving trend in drug delivery is the use of minimally invasive, injectable drug delivery strategies where naturally derived polymers have shown some therapeutic benefit on their own. One such drug delivery system is the hydrogel HAMC, a physical blend of hyaluronan (HA) and methylcellulose (MC). Proteins diffuse readily through HAMC, which also degrades over time [11]. Until now, the use of HAMC has been restricted to delivery of therapeutic molecules into the spinal cords of injured rats [12,13], but its capacity for sustained, localized release makes it an attractive candidate to deliver lymphangiogenic factors as well.

In this study, we report that the delivery of VEGF-C and ANG-2 from HAMC into the nodal excision site has a positive impact on lymphatic function and reduces the magnitude of edema.

## Materials and methods

A total of 60 randomly bred female Dorset sheep (24-45 kg) were used in this study. Animals were given free access to food and water for an observation period of 1 week preceding surgery. To prevent infection, duplocillin was administered intramuscularly 1 day prior to surgery and 2 days postoperatively, and subcutaneous or intramuscular injections of bupernorphine were given postoperatively for pain management. All animal experiments were approved by the animal care committee at Sunnybrook Health Sciences Centre and conformed to the guidelines set by the Canadian Council on Animal Care and the Animals for Research Act of Ontario. All media and reagents were purchased from Sigma Aldrich (Ontario, Canada) unless otherwise stated. VEGFC156S was utilized exclusively throughout this study and is referred to as VEGF-C throughout the remainder of the article.

### Formation and sterilization of HAMC

The formulation and sterilization of HAMC was modified from Gupta's description [11]. To prepare the HAMC components, a 0.5 wt/vol solution of methylcellulose (MC; M7140) in distilled water was created using a dispersion technique. Briefly, MC was added to one third of distilled water at boiling, and once completely wetted, the remaining two thirds of distilled water (cold) were added. The solution was placed in 4°C until completely dissolved. Sodium hyaluronate (HA; molecular weight 1500 kDa, NovaMatrix, Akershus, Norway) was dissolved in distilled water to obtain a 0.1 wt/vol solution. Both solutions were sterile filtered through a 0.22-μm polyethersulfone membrane filtration system (Nalgene, New York, USA) and lyophilized under sterile conditions. To produce growth factor-supplemented HAMC, 3 μg/ml of VEGF-C (R&D Systems, Ontario, Canada) and ANG-2 (R&D Systems) were combined with sterile filtered phosphate-buffered saline (PBS) in a laminar flow hood and mixed to ensure homogeneous dispersion of proteins within the polymer matrix. Methylcellulose was then added, and the mixture was vortexed to dissolve the MC. Last, hyaluronan was added and the solution was vortexed and incubated overnight at 4°C to allow HA to dissolve into the MC solution. Final concentrations of either 1 or 3 μg/ml of growth factors were used throughout the study as indicated.

### Release rates

To determine the release profile of the growth factors from HAMC, 1 ml of growth factor-infused HAMC (1 or 3 μg/ml) was loaded manually into a 3-ml tuberculin syringe. The gel solution was then injected into 9 ml of Dulbecco's modified Eagle's medium (DMEM) supplemented with 0.5% fetal bovine serum (FBS) in a 15-ml Falcon tube. Growth factor release was assessed at 37°C over a 256-hr time period. At each time point, all media were removed and stored at -20°C for further analysis by enzyme-linked immunosorbent assay (ELISA; R&D Systems). Fresh media were then added to each tube, and the experiment was continued. As an internal control, 1 μg/ml and 3 μg/ml samples of each growth factor (in 0.5% FBS-supplemented media) were kept at 37°C for 256 hr.

### Release rate in presence of hyaluronidase

The above release rate experiment was repeated in the presence of the hyaluronan degrading enzyme hyaluronidase (0.5 U/ml to 50 U/ml) in PBS at pH 5.5, the optimum pH for this enzyme (HAase; bovine testicular hyaluronidase).

### Cell culture of bovine lymphatic endothelial cells

The primary bovine lymphatic endothelial cells utilized in this study were generously provided by Dr. Dumont (Sunnybrook Health Science Center, Ontario, Canada). Cultures were maintained in 10% FBS in DMEM on uncoated plates at 37°C and 5% CO_2_. Cultures were supplemented with 10 n/g of VEGF-C to stimulate cell growth. Once cultures reached 70% confluency, they were used in bioactivity experiments.

### Bioactivity of VEGF-C156S and Ang-2 released from HAMC

To ensure that the concentrations of VEGF-C and Ang-2 introduced into the drug delivery system were capable of activating target receptors in lymphatic endothelial cells (LEC), we performed the following experiments. Bovine LEC cultures were serum-starved overnight in DMEM supplement with 0.5% FBS to minimize signaling through the lymphangiogenic pathways of interest. Following the starvation period, cultures were stimulated in one of two ways. In the case of Western blot analysis of VEGFR-3, HAMC (containing 3 μg/mL of both VEGF-C and ANG-2) was added directly onto the bovine plates, and cultures were stimulated for 24 hr. To further reduce background signaling through the Tie-2 receptor, cultures were incubated with release media for 10 min. To form this release media, VEGF-C and ANG-2 (loaded at 3 μg/mL) were released from HAMC for 8 hr, and then the resultant supernatant was centrifuged to remove HAMC. As controls, cultures were also treated with VEGF-C (200 ng/ml) and ANG-2 (800 ng/ml) in 5 ml of 0.5% FBS in DMEM to determine the baseline signaling levels through each receptor. Furthermore, 1 ml of HAMC not loaded with growth factors was included as a vehicle control. Following treatment, cells were lysed for 30 min in phospholipase C-γ lysis buffer (50 mM HEPES (4-(2-hydroxyethyl)-1-piperazineethanesulfonic acid) pH 7.5, 150 mM sodium chloride, 10% glycerol, 1% Triton X-100, 1 mM ethylene glycol tetraacetic acid, 1.5 mM magnesium chloride, 10 mM sodium fluoride, 10 mM sodium pyrophosphate, 1 mM sodium orthovanadate and protease inhibitor cocktail (Roche Diagnostics, DE, USA) for 30 min on ice, centrifuged, and the supernatants of each sample were collected and then immunoprecipitated for VEGFR-3 (C-20, SC-321, rabbit polyclonal antibody; Santa Cruz Biotechnology, Santa Cruz, CA, USA) and Tie-2 (C-20, SC-324, rabbit polyclonal antibody; Santa Cruz Biotechnology) overnight at 4°C. Samples were resolved on 8% sodium dodecyl sulfate polyacrylamide gel electrophoresis (SDS-PAGE) gels and transferred to a polyvinylidene fluoride membrane (GE Healthcare, Ontario, Canada). Blots were blocked in either 5% BSA or 3% milk overnight. The following day samples were incubated in primary antibody (1:1000 anti-phosphotyrosine 4G10; Millipore, Ontario, Canada), Tie-2 and VEGFR3 at 4°C overnight. Blots were then incubated in a horseradish peroxidase-linked secondary (1:10,000, Bio-Rad, Ontario, Canada) for 45 min at room temperature and developed using a chemiluminescence kit (Supersignal West Pico; Thermo Scientific, Ontario, Canada).

### *In vivo *biocompatibility of HAMC

Sheep were fasted 24 hr prior to anesthesia induction. The animals were anesthetized initially by 15-ml intravenous injection of sodium pentothal. Subsequently, 2.0-3.5% isofluorane was delivered through an endotracheal tube via a Moduflex Dispomed machine with Hallowell respirator for surgical maintenance. The dorsal surface of each animal was shaved, washed with soap and water and prepped with alcohol to remove all existing contaminants from skin. Intradermal injections of 100 μl of HAMC and HAMC dually infused with VEGF-C and ANG-2 were administered through 25-gauge needles. Equal volumes of saline and bradykinin (1 mg/ml) served, respectively, as the negative and positive controls. Injections were administered in duplicate once hourly for the first 3 hr (255, 195, 135, 75 min) and then at 45, 30 and 15 min. Following injections at 15 min, 100 μL of ^125^I bovine serum albumin (^125^I-BSA) (0.364 mg/ml; Perkin Elmer, Ontario, Canada) and 25 ml of Evans blue dye (EB) were introduced through a catheter in the cephalic vein, following which the catheter was flushed with 5 ml of heparinized saline. A 15-min time period was then allowed to elapse to allow radioactivity and dye to accumulate at points of inflammation, at which point the animal was killed. Skin encompassing the injection points was removed and photographed with a Nikon digital camera (model CoolPix 4500). Each injection area was then punched out individually using a 2.54-cm cork borer, placed in 10% formalin and measured for radioactivity in a gamma counter (1282 Compugamma CS Universal gamma counter; LLK Wallas, Turku, Finland) to quantify ^125^I-BSA accumulation. Hematoxylin and eosin staining was performed on 7-μm paraffin-embedded cross-sections of the injection areas to determine neutrophil infiltration. On the basis of preliminary results, it became necessary to assess inflammation induction over a longer period of time, and as a result intradermal injections of HAMC (with and without growth factors) were introduced into nonanesthetized sheep at time points corresponding to 24 and 128 hr prior to being killed.

### Induction of lymphedema and growth factor therapy

Lymphedema induction was conducted as previously described by Tobbia *et al*. [2,7]. Fasting and anesthesia induction were performed as described in the previous section. Surgical areas were shaved, washed with soap and water and disinfected with 70% alcohol. A vertical incision (6-8 cm) was made over the lateral aspect of the popliteal region in the hindlimb and the popliteal fossa was exposed. Following identification of the popliteal node in its surrounding fat pad, the pre- and postnodal lymphatics were ligated and the node was excised. At this time, growth factor therapy was introduced. Pre-prepared HAMC (1 ml preloaded into a 3-ml syringe with a blunted 18-gauge needle) was introduced into the area of nodal excision. To increase the retention of HAMC at this site, the fat pad pocket was then carefully sealed with absorbable suture. In the case of sham surgeries, the popliteal fat pad was exposed but undisturbed. A piece of saline-soaked gauze was applied to the area for 10 min to duplicate the length of time needed to perform the growth factor treatment surgery. The animals were returned to their holding pens after recovery from the anesthetic.

### Edema assessment

The hind legs were shaved and leg circumference measurements were taken at a point 10 cm distal to the hock (tarsus). This landmark was highlighted with a skin marker for subsequent measurements postoperatively at the same location. The limb circumferences were divided by the original (presurgical value) and expressed as percentage change over time. To compare the edema outcomes in the various groups, the percentage change in limb circumference was plotted against time, and graphical integration of the area under the curves was calculated using the trapezoidal rule.

### Quantitative assessment of lymphatic function

The primary role of the lymphatic system is to transport protein and fluid from the interstitium back to the venous system. Therefore, the ability to monitor the transport of a radiolabeled protein through the lymphatic system and its accumulation in blood is a measure of lymphatic functionality. This method has been used in several previous studies by our group [2,7]. All animals were fasted, and anesthetic was induced as described above. Evans blue dye (1% in saline) was injected subcutaneously above the hind leg hoof to enhance visualization of the prenodal lymphatic vessels. An incision was made through the skin and subcutaneous tissues over the lower lateral aspect of the hindlimb, and a single vessel was cannulated with a 26-gauge angiocatheter. Saline (100 μl) was injected into the cannula over 30 seconds to check for leaks. Radiolabeled BSA (^125^I-BSA, 0.364 mg/ml, in a 100-μl volume) was then injected over a 60-second period into the prenodal lymphatic vessel and flushed with 100 μl of saline. Blood samples were taken from a neckline inserted into the jugular vein at time 0, and every 5 min up to 1 hr and then every 30 min up to 2 hr. Fluoroscopic imaging was then conducted and the animals were killed (20 ml euthanyl, 240 mg/ml, administered intravenously). It was determined that our radiolabeled tracer was undergoing some breakdown upon introduction into the animal. To determine the extent of the breakdown, trichloroacetic acid precipitations were performed on 2-hr plasma samples from each animal. Supernatant and pellet samples were compared to equivalent volumes of unprecipitated plasma to determine the amount of bound iodine. Plasma samples (1 ml) were then placed in the external gamma counter to determine ^125^I-BSA accumulations in blood over time. These values were expressed as a percentage of the total value of radioactivity (counts per minute) injected and corrected to reflect only BSA still bound to its radiolabel. These values were then plotted versus time. To allow comparison between the experimental groups, the graphical integration of the area under each curve was calculated using the trapezoidal rule.

### Fluoroscopy

Immediately following the lymphatic function studies, imaging was performed. Approximately 1 mL of lipiodol (Ultrafluid; E-Z-EM Canada, Inc., Anjou, Quebec, Canada) was injected into the angiocatheter placed in the upstream popliteal prenodal vessel and the lymphatics/nodes were visualized using a mobile fluoroscopy system (BV Pulsera; Philips Healthcare, Andover, MA, USA).

### Statistical analysis

All data are expressed as means ± SEM unless otherwise stated. The data were analyzed with linear regression or one-way ANOVA followed by a one-sided Dunnett's *t*-test where indicated. We considered *P *< 0.05 to be significant.

## Results

### Release rates

#### Release rates in static cultures

**Figure 1:**
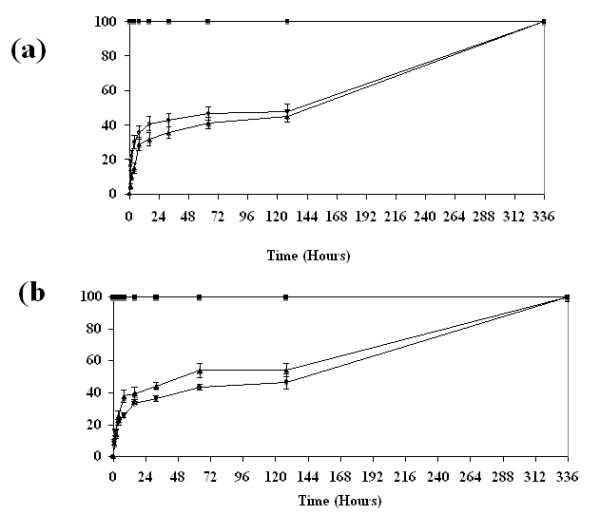
**Percentage cumulative release of angiotensin-2 (ANG-2) and vascular endothelial growth factor (VEGF-C) from HAMC**. **(a) **VEGF-C and **(b) **ANG-2 display similar release profiles in which approximately 50% of initially loaded protein (1 or 3 μg) is released into the surrounding media in the first 32 hr. Following this initial diffusion, controlled phase release rates then plateau. Complete dissolution of HAMC (128 hr) increases recovery of both factors to 100%, suggesting that matrix degradation also plays a role in release. Data are shown as means ± SEM (*n *= 3). Control (square), 1 μg (triangle), 3 μg (circle).

Figure 1 displays cumulative release rate profiles in which each VEGF-C (Figure 1a) and ANG-2 (Figure 1b) (1 or 3 μg/ml) is continuously released from HAMC over a period of 128 hr. Within this time, growth factor recovery was approximately 50%, with the majority of releases occurring by hour 32, indicating that about half of the initially loaded protein can be released from HAMC through diffusion. These results correspond to those previously reported for release profiles of growth factors from HAMC [11,13]. When HAMC had completely degraded/dissolved into the media over the 2-week period, growth factor recovery was approximately 100%, suggesting that matrix degradation may be needed for complete growth factor release.

#### Release rate in the presences of Hyaluronidase

**Figure 2:**
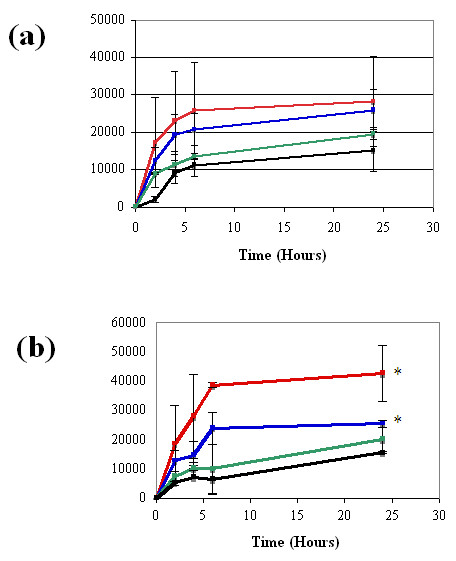
**Hyaluronidase increases the release rate of VEGF-C and ANG-2**. Increasing levels of hyaluronidase activity (U/ml) is accompanied by increasing release of **(a) **VEGF-C or **(b) **ANG-2 from HAMC. Data are shown as means ± SEM (*n *= 3). Fifty units (red), 5 U (blue), 0.5 U (green), and no enzyme (black) are shown. Significant differences in ANG-2 release were reached by hour 24 in both the 50 U and 5 U enzyme concentration solutions. VEGF-C release did not reach significance. Significance in comparison to control levels (no HAase) was assessed by ANOVA and Dunnett's one-sided *t*-test and is indicated by asterisks. *P < 0.05.

Hyaluronidase (HAase) is one of the primary enzymes involved in hyaluronan degradation, cleaving the β1-4 glycosidic bonds of this protein. This enzyme can be found both on the cell surface (HYAL2) and within the lysosome (HYAL1), and high levels of this enzyme are often associated with high levels of hylauronan turnover [14,15]. Since it was unknown whether the popliteal fat pad would contain relatively low or high levels of HAase, a dilution series of HAase activity levels was used to assess the potential effect this enzyme could have on release rates from HAMC. The activity level, 50 U, is representative of the highest natural tissue level of this enzyme found within the human body (liver). In the presence of hyaluronidase, HAMC displayed dose-dependent release of growth factors that correlated with increasing hyaluronidase concentration (Figure 2). More ANG-2 was released in the presence of hyaluronidase (50 and 5 U) in comparison to controls (no HAase), reaching significance at 24 hr. While a similar trend is observed for VEGF-C release in the presences of HAase, groups were not significantly different, most likely due to increased growth factor instability at the low pH used. Ultimately, this experiment revealed that the presence of hyaluronidase at the site of HAMC injection will likely influence growth factor release.

### Bioactivity

#### Sequence homologies

It was necessary to determine whether the growth factors released from HAMC remained bioactive, that is, could bind to their target receptors and activate their respective signaling cascades in sheep. The growth factors employed in this study are human derived and thus share only partial homology with those of sheep. Specifically, human ANG-2 protein shares 89.6% homology with its sheep counterpart (HomoloGene, NCBI). While no data were available for ovine VEGF-C, it was determined that bovines (also a ruminant animal) and humans share 88.3% homology (HomoloGene, NCBI). The receptors for each of these ligands share similar homologies at 86.8% for VEGFR-3 (between bovine and human) and 95.4% for Tie-2 (between bovine and human) (HomoloGene, NCBI). The homologies appear to be sufficiently similar that one can expect reactivity between the sheep receptors and their human ligands.

#### Stimulation of LEC cultures

**Figure 3:**
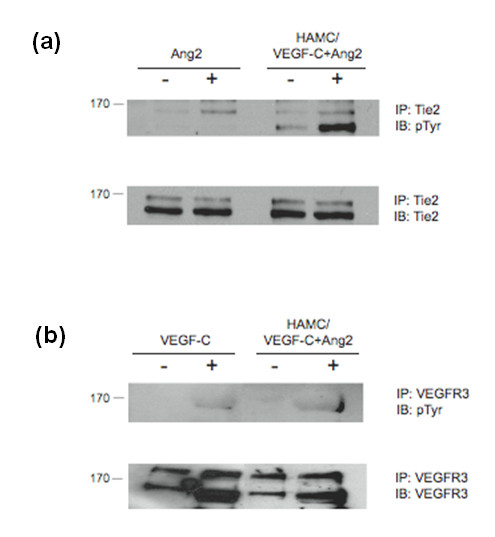
**Growth factors released from HAMC possess the ability to stimulate lymphatic endothelial cells**. Purified bovine lymphatic endothelial cells (LECs) were stimulated with conditioned media from HAMC alone or HAMC infused with ANG-2 and VEGF-C. As controls, cultures were also treated with VEGF-C156S (200 ng/ml) and ANG-2 (800 ng/ml). Immunoprecipitated **(a) **Tie-2 or **(b) **VEGFR-3 activation was assessed by phosphotyrosine (pTyr). Cultures treated with growth factor release media displayed increases in receptor phosphorylation levels in comparison to negative controls (unstimulated and HAMC alone). Blots were stripped and reprobed to confirm similar receptor levels across treatment groups. *n *= 2. Molecular weights of each receptor are indicated.

On lymphatic endothelial cells, ANG-2 acts through the receptor Tie-2, while VEGF-C165S is known to associate exclusively with the receptor VEGFR3 [16]. Lymphatic endothelial cell cultures [17] were stimulated with conditioned media from either HAMC alone or HAMC infused with ANG-2 and VEGF-C. Significantly, HAMC infused with ANG-2 and VEGF-C induced an increase in cognate receptor phosphorylation over HAMC conditioned media alone (Figure 3). Interestingly, HAMC conditioned media alone can induce receptor activation of Tie-2, although levels did not reach those induced by ANG-2. These results demonstrate that both ANG-2 and VEGF-C, when released from the HAMC vehicle, can activate their cognate receptors *in vitro*.

### Biocompatibility

Previous studies have shown that HAMC attenuates the inflammatory response and promotes healing of the dura when injected into the intrathecal space in either control or spinal cord injury rats [11]. We felt it necessary to determine whether similar results could be expected upon introduction of growth factor-infused HAMC in our sheep model.

#### Qualitative and quantitative assessment of vascular permeability: Evans Blue and ^125^I-BSA study

**Figure 4:**
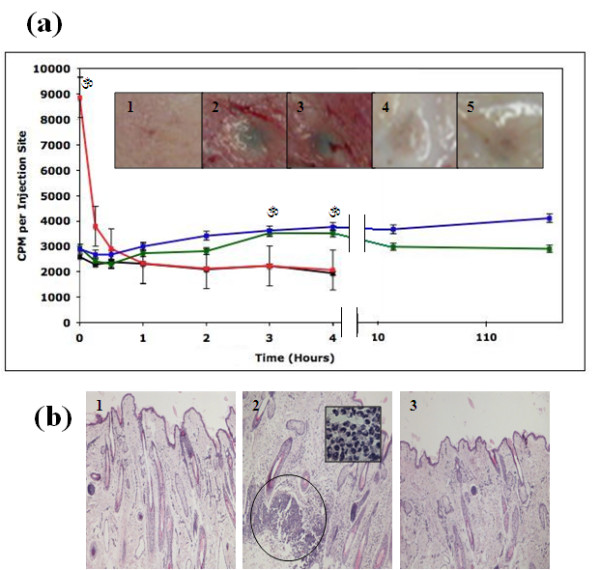
**HAMC induces minimal inflammation in sheep**. Introduction of HAMC (HAMC alone (green)) and HAMC infused with 3 μg/ml of ANG-2 and VEGF-C (blue) resulted in an increased accumulation of **(a) **^125^iodine radiolabeled bovine serum albumin (^125^I-BSA) in comparison to saline (black) injections at corresponding time points. These vascular permeability increases reached significance at 3 and 4 hr postinjection; however, they appeared to resolve over a 1-wk period. Bradykinin, a transient vascular permeability inducer, was used as a positive control (red). Data are shown as means ± SEM (0-4 hr, *n *= 5; 24 and 168 hr, *n *= 2). Double lines indicate a change in scale. Significance in comparison to saline was assessed by ANOVA with accompanying one-way Dunnett's *t*-test. *P < 0.05. Similar results were obtained when Evans blue dye (EB) accumulation was examined ((a) *inset*), saline 4 hr (1), HAMC 4 hr (2) HAMC + GFs 4 hr (3), HAMC 1 wk (4), HAMC + GF 1 wk (5). **(b) **Hematoxylin and eosin-stained skin sections of injection areas were examined using a bright-field microscope at ×10 magnification. There were areas of heavy neutrophil infiltration in HAMC injection areas at 4 hr (2) in comparison to saline controls (1). Neutrophil infiltrations appeared to resolve over the next week in HAMC injection areas (3). Circle indicates an area of heavy neutrophil infiltration. *Inset*: Enlargement of cell infiltration area depicting classical morphology of neutrophilic cells, ×100 magnification.

An effective method to measure the induction of inflammation is to quantify changes in vascular permeability. This is achieved by monitoring the accumulation of intravenously administered dye or radiolabeled protein at the site of interest. In this study, we employed the use of EB and ^125^I-BSA accumulation following interdermal injections of HAMC. After sheep were killed, injection areas were first examined for EB accumulation. The outer and undersurface of the skin displayed slight blue staining corresponding to HAMC injection areas at 1-4 hr (Figure 4a, *inset*). This color development was greater than that exhibited by the saline controls (no color), but not reaching the blue staining level visualized in the bradykinin injections points at 0 min and 15 min. Injection areas corresponding to 1 day and 1 wk did not show any dye accumulation, suggesting that vascular permeability had subsided by these time points (Figure 4a, *inset*).

Next, analysis at injection was conducted for ^125^I-BSA. These results suggested that HAMC (regardless of growth factor presence) induced a minimal level of inflammation. Levels of radioactive BSA were approximately double those observed in saline-injected sites but never reached the levels attained by bradykinin injection (Figure 4a). Over a 1-wk period, the skin BSA levels induced by HAMC remained relatively constant and elevated in comparison to saline controls (only reaching significance at the third and fourth hours following introduction). We concluded that HAMC did induce some transient inflammation over the monitoring period.

#### Histology

An increase in the presence of neutrophils was observed compared to control injections at hour 4 (Figure 4b). However, by the end of week 1, HAMC injection areas resembled those of controls. This result suggested that the mild inflammation induced by HAMC was temporary.

### Lymphatic transport function

**Figure 5:**
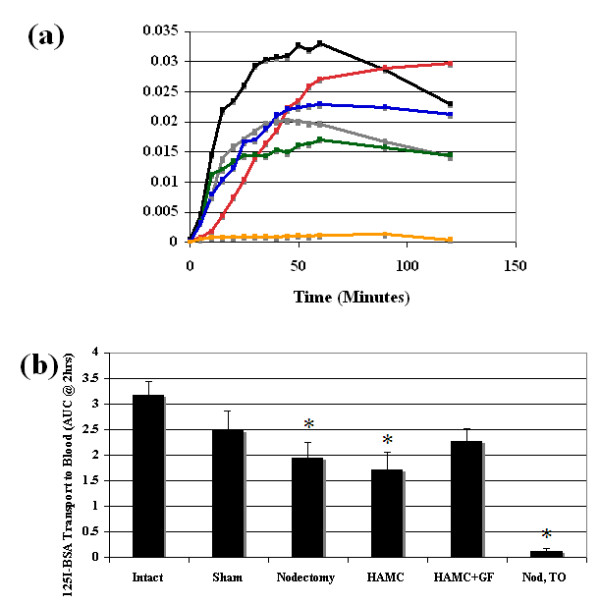
**Lymphatic functionality**. **(a) **Removal of the popliteal lymph node results in lymphatic vessel damage and immediate assessment (Nod T0) reveals that minimal ^125^I-BSA transport to plasma occurs (orange, *n *= 7). Growth factor treatment (blue, *n *= 8) at the time of node removal resulted in an increase in ^125^I-BSA accumulation levels in plasma over 2 hr in comparison to nontreated groups, nodectomy (gray, *n *= 8) and HAMC (green, *n *= 6). Growth factor treatment accumulation levels did not return to those seen in sham (red, *n *= 7) and intact control groups (black, *n *= 7). Points represent means. SEM was omitted for clarity. **(b) **The area under each curve was then tabulated for each individual animal using the trapezoidal rule. Following node removal, nontreated groups (nodectomy, HAMC, Nod T0) had significantly less functionality at 6 wk in comparison to the intact group. In contrast, functionality in the growth factor treatment group was similar to the intact group. Each bar represents mean ± SEM. Significance in comparison to control levels (intact limbs) was assessed by ANOVA and Dunnett's one sided *t*-test. *P < 0.05. BSA, bovine serum albumin.

The average tracer accumulation in blood is illustrated for all groups in Figure 5a. The black line (intact limbs) provides the reference point for the various interventions. At the other extreme (orange line), very little BSA enters the blood immediately after lymph node excision, because the lymphatics have been disrupted. It is clear that sham surgical procedures impact lymphatic function negatively, presumably because some of the fragile vessels have been damaged (red line). By 6 wk after excision, the normal lymphatic regeneration has improved lymph transport somewhat (gray line) and this is similar to that observed with HAMC alone (green line). Some improvement in lymphatic function was noted in the group receiving HAMC plus growth factors (blue line).

To determine whether the functionality changes were significant, the areas under the curves were compared. Lymph node removal was found to significantly reduce functionality in the nontreated groups (nodectomy 1.9 ± 0.9 or HAMC alone 1.7 ± 0.8) in comparison to intact controls (3.2 ± 0.7) (Figure 5b). Alternatively, values for the growth factor-treated groups (2.3 ± 0.7) were not significantly different from the values observed in both sham and control groups. This indicates similar lymphatic functionality across all three groups (growth factor-treated, sham and intact). Growth factor-treated limbs did not return completely to the functionality levels measured in intact limbs over the 6-wk period.

### Fluoroscopy

**Figure 6:**
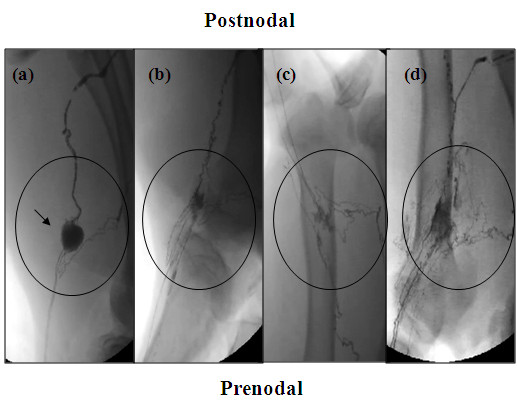
**Fluoroscopy of popliteal fossa region**. Fluoroscopic images of the lymphatic networks 6 wk following surgery were taken. Representative images from each group are shown above. In sham preparation **(a) **at 6 wk, the popliteal lymph node (arrow) is visible as well as several pre- and postnodal lymphatics. In the cases where nodes were removed (nodectomy **(b) **and HAMC **(c)**), at 6 wk a tangled network of lymphatics is visible at the surgical site, suggesting some lymph continuity has been reestablished through natural lymphangiogenesis. Growth factor-treated animals **(d) **displayed an increase in the number of vessels in the excision site. This increase in vessels may be responsible for the increase in lymphatic functionality seen in this group. Circles indicate popliteal fossa region.

In intact and sham surgical groups, there were clearly visible popliteal lymph nodes with identifiable prenodal and postnodal vessels (Figure 6a). Six weeks after lymph node excision, some fluid continuity had been reestablished, because both the afferent and efferent vessels were visible and connected by an entanglement of newly regenerated vessels (example in Figure 6b). These newly formed vessels appeared to be fragile as indicated by the frequent leaks in this area upon contrast agent infusion. The irregular regeneration pattern and what appeared to be partial filling of some of the vessels precluded meaningful quantitative analysis between groups. However, general trends were noted. In the HAMC alone group, the regeneration pattern was similar to that observed in the nodectomy group (example in Figure 6c). In the limbs receiving HAMC plus growth factors, there appeared to be an increase in the number of regenerated vessels (Figure 6d).

### Edema

**Figure 7:**
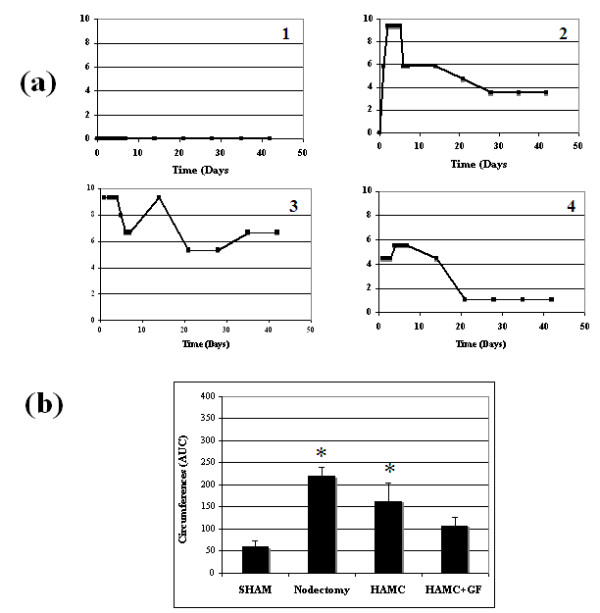
**Development of edema following popliteal lymph node excision in the presence and absence of growth factor therapy**. **(a) **Typical examples of edema formation in each treatment group (*n *= 1) are displayed. **(b) **The area under the curve (AUC) was calculated for animals using the trapezoidal rule. Node removal (nodectomy (2)) and node removal with HAMC (3) usually resulted in a sharp increase in edema formation over the first week and decreased marginally over the next 5 wk. In contrast, growth factor-treated groups displayed reduced edema levels by 6 wk (4), statistically similar to sham levels (1). Each bar represents mean ± SEM. Sham *n *= 18, nodectomy *n *= 36, HAMC *n *= 7, HAMC + GF *n *= 7. Significance in comparison to sham levels was assessed by ANOVA and Dunnett's one sided *t*-test. *P < 0.05.

As expected, limb circumferences did not change over time in the sham-treated animals. In contrast, the removal of a popliteal lymph node resulted in edema formation in 100% of animals. The presence of HAMC plus growth factors appeared to produce the best results in terms of reducing edema levels. Typical examples from each group are illustrated in Figure 7a.

Similar to the lymphatic functionality experiment, the areas under the curves were used to conduct quantitative comparisons between groups (Figure 7b). Growth factor-treated groups displayed edema magnitudes slightly greater than but not significantly different to sham levels (107.4 ± 51.3 and 59.2 ± 62.5, respectively). Nontreated groups displayed significantly greater edema levels in comparison to the sham group (nodectomy 219.8 ± 118.7 and HAMC 162.6 ± 141). This result suggests that growth factor treatment has the ability to reduce edema magnitude following lymph node removal.

**Figure 8:**
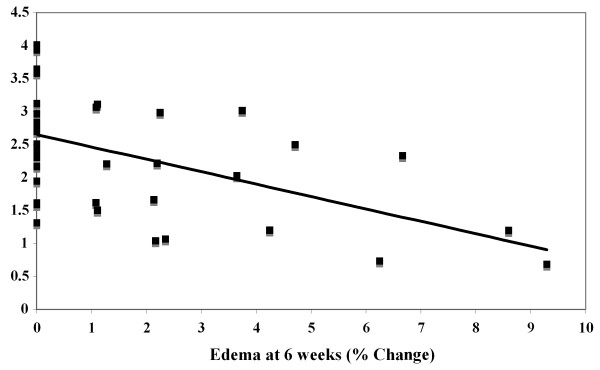
**Relationship between lymphatic functionality and edema magnitude**. Functionality data (AUC at 2 hr) were plotted versus their corresponding edema values at 6 wk, *n *= 36. The trend shows a negative correlation, suggesting that as lymphatic functionality increases, edema magnitude at 6 wk decreases. Linear regression analysis revealed this trend to be significant, *P *= 0.001.

When we plotted all of the individual lymphatic function data versus their corresponding edema measurements at 6 wk for all groups, we observed a significant negative correlation (*P *= 0.001) (Figure 8). That is, the highest levels of lymphatic function correlated with the lowest levels of edema, supporting the notion that improvement in lymph transport capacity with growth factors has potential for lymphedema treatment.

## Discussion

In this study, we report that the addition of two lymphangiogenic growth factors to a nodal excision site enhances lymphatic transport function and reduces the edema burden in a sheep model of lymphedema. These data suggest that molecular therapeutic approaches may ultimately be effective in improving outcomes for breast cancer patients with lymphedema. For this concept to be valid, some discussion of animal models, choice of molecules and drug delivery systems and comparisons with other potential therapeutic approaches are warranted.

### Choice of animal model for investigation of lymphedema

It is clear that new animal models need to be developed to understand more fully the pathogenesis of lymphedema and to develop new therapeutic approaches [18]. The use of small animals, especially the mouse, has been very beneficial in helping to elucidate the molecules that play a role in lymphangiogenesis. However, a major advantage of utilizing a larger animal, such as the sheep, for lymphedema studies is that lymphatic function can be measured, something that is extremely difficult to achieve in a small species. In sheep, the pre- and postnodal popliteal ducts are easy to identify, and some of these are of sufficient size for cannulation. In this regard, the ability to transport a known mass of radiolabeled albumin to plasma provides a useful measure of the lymph transport effectiveness of a given lymphatic network. Therefore, following the injection of ^125^I-BSA into prenodal lymphatic vessels, one would expect that the mass transport rate of this protein tracer to plasma would reflect the integrity of the lymphatic network in question, a notion that is supported by the data in this report.

In humans, lymph-venous anastomoses can form with long-term lymphatic obstruction [19,20]. If such connections were to exist in our sheep experiments, the labeled BSA would be diverted into the limb vasculature and would not pass through the nodal excision site. However, over the many years we have used the sheep model, we have never observed these connections in the popliteal region. Presumably, the level of impediment caused by nodal excision in the animal model is relatively benign compared to that which occurs in humans with cancer. In the human case, the surgical procedures related to the removal of the cancer and lymph nodes are much more extensive and likely provide a significantly greater insult to the lymphatic system than that occurring in our experiments. In the latter case, relatively robust lymphangiogenesis occurs and some degree of fluid continuity is restored relatively quickly so that the stimulus for lymph-venous connections in the leg may not be as great as is the case with some lymphedema patients.

In cancer patients, the removal of one or more lymph nodes often gives rise to acute edema which resolves successfully. It is, of course, the chronic form of edema that occurs in a subgroup of patients that is most problematic. The exact conditions that drive the formation of this subgroup are unknown. It has been suggested that women with greater than average peripheral lymph flow are more likely to develop lymphedema following breast cancer surgery [21]. The authors postulated that this predisposing factor might explain why lymphedema develops in patients in which only a few nodes have been removed. In all likelihood, lymphedema is the result of a complex interplay between many factors (surgery, lymph node removal, radiation, injury and predisposing characteristics) that lead to the development of this condition. It should be noted that we are not attempting to replicate the complex conditions experienced by cancer patients in our model. Instead, our model permits us to examine the properties of a single lymphatic network in order to develop basic therapeutic principles that could be applicable to the human condition.

There is no agreed-upon time beyond which one considers edema to be chronic. For practical reasons, we did not follow the animals beyond a 6-wk period. Nonetheless, we feel that our sheep model is suitable for the measurement of lymphatic-related physiological changes over time and can provide a realistic framework for the development of therapeutic measures and methods of administration that are human-sized in perspective.

### Drug delivery systems

There are many potential drug delivery approaches that could be used to introduce lymphangiogenic growth factors. The HAMC system offers several advantages in this regard. A hydrogel represents a network of water-swollen polymer chains in which water is the dispersion medium. When HAMC is placed within the body, it absorbs water from the surrounding environment, the pore sizes within the matrix expand and proteins are released [22]. This characteristic property of hydrogels accounts for the rapid burst release of growth factors at the beginning of each release study. In the case of our system, following this initial rapid release, rates appeared to slow until HAMC had undergone dissolution. This suggests that growth factors incorporated into the outer regions of HAMC could readily diffuse into media, but the diffusion distance may have been too great for those incorporated into the core of HAMC, causing release to plateau. Degradation of HAMC then allows for the release of remaining factors.

Previous HAMC studies saw almost complete diffusion-mediated release of a single growth factor from this system over a 16-hr period [13], but in these experiments the volume of HAMC used was 1000-fold less than the volumes utilized in our report.

Hyaluronan is metabolized by hyaluronidase *in vivo*, and it has already been shown that this enzyme can affect the degradation of HAMC [11]. As anticipated, increasing activity levels of HAase in our study were associated with increasing release rates of both growth factors. This result suggests that endogenous HAase activity in the popliteal fossa may result in more rapid release of lymphangiogenic factors *in vivo *than predicted from the *in vitro *experiments. While we do not know the release profiles of VEGF-C and ANG-2 *in vivo*, we expect that the release rates will depend upon consumption of the factors (maintaining a concentration gradient favoring release) in the popliteal fossa and the presence of degrading enzymes, such as hyaluronidase. We anticipate that the bioactivity of released factors will be maintained over the course of the release, owing to their incorporation into HAMC. The half-life of VEGF-C in the circulation is less than 15 min; thus HAMC will likely provide some protection to the growth factors by controlling its release over time [23].

The HAMC drug delivery system contains numerous components that could potentially invoke a reaction from the host: the methylcellulose is synthetically manufactured, hyaluronan is produced in bacteria and the growth factors are all human derived. Nonetheless, the evidence presented in this report, including assessments of vascular permeability and leukocyte infiltration, indicates that any host response is minimal. This is supported by the literature where HA has been shown to attenuate the inflammatory response in several tissues [24].

### Choice of growth factors and in vivo bioactivity

One of the most important questions in devising a molecular strategy for lymphedema treatment is the selection of the lymphangiogenic growth factor. In this regard, VEGF-C seemed an obvious choice. VEGF-C is required for the development of the lymphatic system as well as for proliferation, migration and survival of LECs [9]. It is also known that blocking signaling through its receptor, VEGFR-3, results in regression of preexisting lymphatic vessels. Recent studies in mice have revealed that VEGF-C introduction increases the rate of damaged vessel reconnection in comparison to controls [25]. After lymph node excision and transplantation, VEGF-C was observed to facilitate vessel connection to the transplanted tissue [26]. Furthermore, evidence has been found that directly links VEGF-C signaling and lymphedema. Point mutations identified in VEGFR-3 lead to a hereditary form of lymphedema, known as Milroy's disease that presents with edema of the lower extremities [27,28].

Another factor we decided to introduce into the lesion site is angiopoietin-2. ANG-2 has been implicated in the organization of the lymphatic system through the activation of the Tie-2 receptor. ANG-2-knockout mice display abnormal lymphatic patterning, disrupted lymphatic endothelial-smooth muscle cell interactions as well as subcutaneous edema and chylous ascites, indicting an obligatory role for ANG-2 in lymphatic system development [10,17].

Overall, this study revealed that both growth factors appear to be protected from inactivation while incorporated in HAMC until their release, when they can in turn bind and activate target receptors involved in lymphangiogenic signaling. Owing to close homology between both bovine and ovine ligands and receptors, these results suggest that similar results would likely be obtained with ovine cells, which was supported by new vessel growth in the fluoroscopic images.

Additionally, we found that HAMC appears to potentiate Tie-2 phosphorylation by ANG-2. Specifically, there appears to be an increase in Tie-2-ANG-2 association demonstrated by the increased presence of a second band in these cultures. As HAMC undergoes dissolution, the viscosity of media surrounding the cells increases, which may alter the interaction of Tie-2 with ANG-2 dimers (which form spontaneously in solution), perhaps increasing their association time, explaining the above result.

This study also indicated that HAMC may possess the surprising ability to activate the receptor tyrosine kinase (RTK) Tie-2. It is well known that low molecular weight hylauronan fragments play a role in endothelial cell proliferation and migration through activation of particular RTKs [29]. Typically, these fragments range from 3 to 16 oligosaccharides in length [30]. However, the hylauronan utilized was initially 1500 kDa, and while small amounts of random cleavage may occur, this seems like an unlikely cause of receptor activation. A second, more plausible theory is that through an association with CD44, hyaluronan indirectly influences Tie-2 signaling. Hylauronan can directly bind CD44, causing the cell surface receptor to cross link [31]. This cross linking has been shown to influence integrin signaling [31], which in turn can influence Tie-2 activation [32]. All three are common receptors found on the surface of endothelial cells, and therefore association with CD44 could indirectly affect Tie-2 signaling. The ability of HAMC to independently activate a RTK such as Tie-2 may provide an additional advantage to the lymphangiogenic process, working synergistically with the ligand ANG-2 to improve functionality.

### Other factors to consider

Our results suggest that prolymphangiogenic growth factors can have a positive impact on lymphatic function and improve edema outcomes. However, there are several conceptual issues that may need refinement in future investigations. First, there is evidence that lymphangiogenesis is a two-phase process. The initial phase involves activation of endothelial cells and sprouting, while the second phase involves maturation of these vessels. Maturation includes smooth muscle cell recruitment, which appears to play an important role in the orientation of newly formed vessels [10]. With this in mind, it may be possible to build a system to control the temporal release of the growth factors to mimic more closely the endogenous realities. It should be noted, however, that the dual factor approach and the doses we utilized in this study had a significant impact on lymphatic function and edema resolution. This information provides a useful framework on which to optimize lymphangiogenic therapeutic approaches in future studies.

A second issue involves the signaling ability of VEGFC156S (utilized here) versus its wild-type counterpart. The wild-type ligand has the ability to bind both VEGFR2 and VEGFR3 receptors, while VEGFC156S is an agonist only to VEGFR3. Signaling through VEGFR2, which is found predominantly on vascular endothelium, causes increased permeability, an event that we wanted to minimize in our study. However, upon initial characterization, it was noted that VEGFC156S signaling in LECs was different from VEGF-C [16]. It was postulated that activation of VEGFR2 on LECs might be needed to induce maximal lymphangiogenic signaling. In any event, it is clear that manipulation of lymphangiogenesis *in vivo *is a complex issue and that optimal conditions (choice of factor, dosage and timing of application) require further investigation.

### Comparison of lymphangiogenic based therapies with other potential approaches

The appeal of lymphangiogenic-based therapies for the treatment of lymphedema is easy to understand. This type of treatment has the potential to reverse the disease state by facilitating naturally occurring processes. However, processes such as lymphangiogenesis are complex and not fully understood yet. Furthermore, while molecular therapies have shown benefits in breast cancer-related lymphedema, the exact nature of the defect that causes lymphedema is still unknown, and therefore the most appropriate approach to the treatment method for this disease must still be determined.

Autologous lymph node transplantation is an alternative experimental treatment for lymphedema. It is well documented that removal of the lymph nodes greatly increases the chance of developing lymphedema, especially if this is combined with radiotherapy [33]. In a previous report, we demonstrated that a successful vascularized popliteal node transplant in sheep could effectively restore lymphatic function and limit or prevent edema development when applied at the time of nodal excision [2]. Indeed, Becker and colleagues [34] transplanted femoral nodes into the axillary region of 24 postmastectomy lymphedema patients using microsurgical procedures followed by manual drainage (physiotherapy) during the first 3 months. Overall, edema resolution was good, especially in those individuals in whom the lymphedema was of the shortest duration. Surgical complexities and potential donor site morbidity issues complicate the application of lymph node transplantation in clinical practice.

## Conclusions

The HAMC drug delivery system has been successfully adapted for the introduction of prolymphangiogenic molecular factors to a nodal excision site. The growth factors employed in this study, VEGF-C and ANG-2, enhanced the naturally occurring lymphangiogenesis, significantly increased lymph transport and reduced the magnitude of edema formation. Results from this study highlight the potential of molecular therapies for lymphedema patients.

## Abbreviations

ANG-2: angiopoitein-2; DMEM: Dulbecco's modified Eagle's medium; EB: Evans blue; ELISA: enzyme-linked immunosorbent assay; FBS: fetal bovine serum; HA: hyaluronan; HAase: hyaluronidase; ^125^I-BSA: ^125^iodine-radiolabeled bovine serum albumin; LEC: lymphatic endothelial cell; MC: methylcellulose; PBS: phosphate-buffered saline; RTK: receptor tyrosine kinase; SDS-PAGE: sodium dodecyl sulfate polyacrylamide gel electrophoresis; Tie-2: tyrosine kinase with immunoglobulin-like and endothelial growth factor-like domain-2; VEGF-C: Vascular endothelial growth factor-C; VEGFR-3: vascular endothelial growth factor receptor 3.

## Competing interests

Dr. Molly Shoichet holds the patent for HAMC, the drug delivery system employed in this study. The other authors declare that they have no competing interests.

## Authors' contributions

AB performed the physiological studies, helped to plan the study and participated in its design and coordination. HK performed the molecular analyses. JS conceived of the study, and participated in its design and coordination. DD participated in the study design and coordination. MS participated in the study design and provided help in the preparation of HAMC. DT participated in the study design. MJ conceived of the study, and participated in its design and coordination.
